# Introducing BioSARN – an ecological niche model refinement tool

**DOI:** 10.1002/ece3.2331

**Published:** 2016-07-22

**Authors:** Marshall J. Heap

**Affiliations:** ^1^ Carl R. Woese Institute for Genomic BiologyUniversity of Illinois at Urbana‐Champaign1206 W Gregory DrUrbanaIllinois61801

**Keywords:** Environmental niche modeling, high spatial resolution, land class model, realized distribution, enhanced temporal corridors

## Abstract

Environmental niche modeling outputs a biological species' *potential* distribution. Further work is needed to arrive at a species' *realized* distribution. The Biological Species Approximate Realized Niche (BioSARN) application provides the ecological modeler with a toolset to refine Environmental niche models (ENMs). These tools include soil and land class filtering, niche area quantification and novelties like enhanced temporal corridor definition, and output to a high spatial resolution land class model. BioSARN is exemplified with a study on Fraser fir, a tree species with strong land class and edaphic correlations. Soil and land class filtering caused the *potential* distribution area to decline 17%. Enhanced temporal corridor definition permitted distinction of current, continuing, and future niches, and thus niche change and movement. Tile quantification analysis provided further corroboration of these trends. BioSARN does not substitute other established ENM methods. Rather, it allows the experimenter to work with their preferred ENM, refining it using their knowledge and experience. Output from lower spatial resolution ENMs to a high spatial resolution land class model is a pseudo high‐resolution result. Still, it maybe the best that can be achieved until wide range high spatial resolution environmental data and accurate high precision species occurrence data become generally available.

## Introduction

Environmental niche modeling (ENM) is a popular approach to ecological niche modeling of biological species (Peterson 2011). Two established ENM tools are MAXENT (Phillips et al. 2004) and openModeller (Sutton et al. 2007). In their study, introducing MAXENT, the authors describe the model output as the species' *potential distribution* that could then be used to estimate the species' *realized distribution* by removing areas where the species is known to be absent (Phillips et al. 2004). Sutton et al. (2007) opt for the term *fundamental niche* to describe the model output of openModeller and other ENMs including MAXENT. Regardless of the terminology used to describe ENM output, there are many constraints on a species *realized* distribution, for example, topography, habitat destruction, anthropogenic land‐use, invasive species, and remnant unreproductive populations (Peterson 2011). These factors may either be unknown to the ENM algorithm or underestimated.

A particular challenge facing experimenters when modeling species distributions over large geographic areas at high spatial resolution is the paucity of both high resolution environmental and species occurrence data. At such scales, the highest resolution climatic data publicly available is 30‐arc second (≈1‐km) data, for example, WorldClim – Global Climate Data (2015) and PRISM Climate Group, Oregon State University (2014). Climatic data are a core ENM requirement, constraining the spatial resolution of the output model. Species decimal degree occurrence data must be stated to at least two decimal places for use with 1‐km environmental data without introducing positional error (Heap and Culham 2010). GBIF (http://www.gbif.org/) is the major online source of these data, but finding temporally and spatially accurate data, there is a challenge. For example, there are 330 GBIF records for *Abies fraseri* (GBIF.org 2016) but only 18 of these relating to the 1980–2010 time frame are spatially precise to within 1 km. By contrast, land‐use data are often available at very high spatial resolution, for example, at 30 m in the case of the NLCD 2011 dataset for the coterminous USA (Homer et al. 2015).

Biological species climate change studies typically involve baseline and projected climate models. A subset of these studies has focused on the development of temporal corridors of environmental continuity (Hamann and Wang 2004, 2006). Rose and Burton (2009) used the “Overlay–Intersect” tool in ArcGIS (http://www.esri.com/software/arcgis) to map such corridors at the same 1‐km resolution of the ENMs. Further computations were required to count the grid cells comprising these corridors and convert them to km^2^. The resultant maps permitted identification of these temporal corridors but neither the direction nor quantification of niche movement.

A fairly recent ecological software innovation is ModEco (Guo and Liu 2010). This tool allows the modeler to mix environmental layers at differing extent and resolution. Output can be at a custom resolution or the minimum/maximum resolution in the environmental group. However, data uncertainty will be introduced where the precision of species occurrence data is less than that required for the chosen output resolution (i.e., the risk that species locations will be matched with incorrect environmental layer values). ModEco offers model suggestions based on occurrence data type (e.g., presence only, presence/absence, and abundance data) but occurrence data precision issues are left to the modeler to recognize.

Consequently, this study's aim was to show how Biological Species Approximate Realized Niche (BioSARN) (https://sourceforge.net/projects/biosarn/) can refine ENMs through feature file filtering, enhanced temporal corridor definition, niche area quantification, and output to a high spatial resolution land class model (LCM).

In order to use BioSARN, the modeler must first construct biological species ENMs using established modeling techniques. Key issues that should be addressed here are species occurrence data cleaning (Heap and Culham 2010; Soley‐Guardia et al. 2016), mitigation of sampling bias (Phillips et al. 2009; Boria et al. 2014), data partitioning/ENM settings (Phillips et al. 2006), and ENM parameterization/evaluation (Muscarella et al. 2014; Soley‐Guardia et al. 2016). Subsequently, the modeler must construct the environmental layers which BioSARN will use to trim the ENM‐generated *potential* distribution to a closer approximation of the species' *realized* distribution. This methodology is illustrated by a worked example preceded by a general description of BioSARN.

## BioSARN

### General description

**Figure 1:**
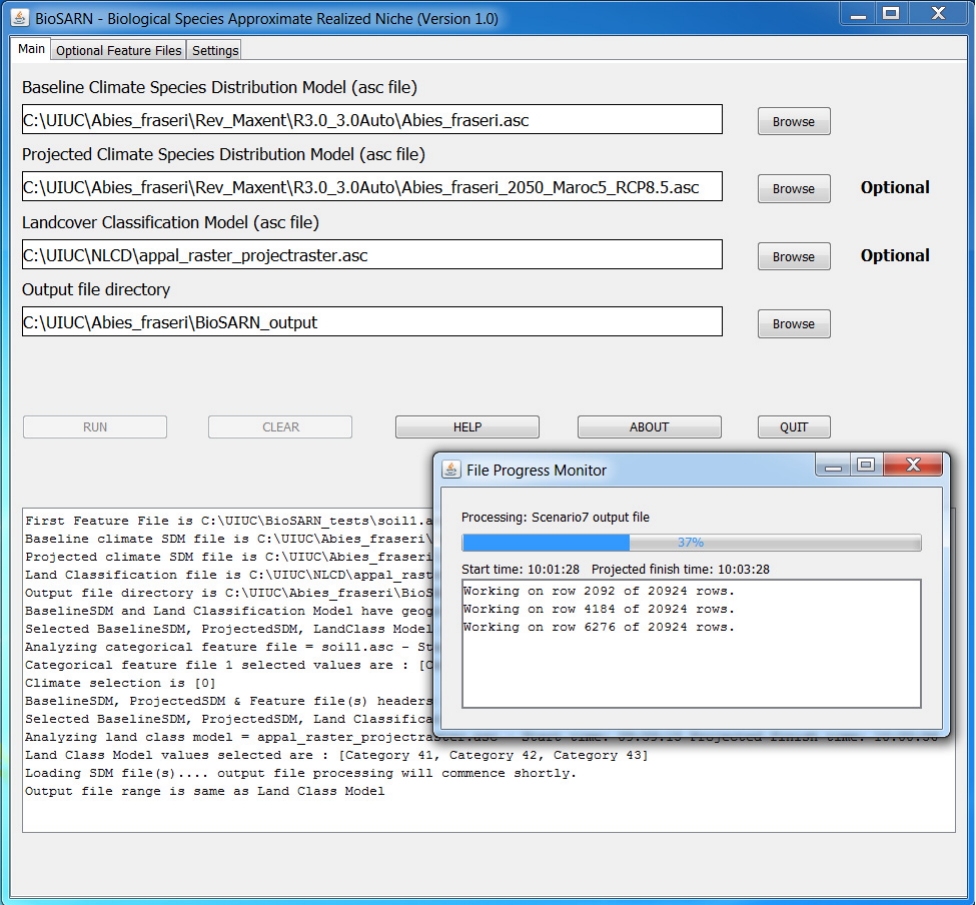
BioSARN “main” screen illustrating *Abies Fraseri* example.

Biological Species Approximate Realized Niche (BioSARN) is a Java^™^ Desktop MicroSoft Windows Application with a simple GUI. Depending on scenario, the user can select a baseline climate ENM (derived from observed temperature and rainfall data), a projected future climate ENM and a LCM on the application's “main” screen (Fig. 1). ENM output can be filtered by up to three feature files (e.g., edaphic and topographic data) on the next screen. Additionally, the user can specify categorical/continuous data format and applicability of feature files to either or both climate scenarios. All input files must be in ASCII format and except the LCM must have identical range and spatial resolution. Use of the ASCII file format renders BioSARN compatible with most commonly used GIS (e.g., ArcGIS, DIVA‐GIS, OpenModeller, and Global Mapper). Environmental suitability thresholds are specified on the “settings” screen. The “RUN” button is highlighted when BioSARN detects a runnable scenario and then inaccessible until run termination and selection of a new scenario. Input and status information is provided to the user via the “main” screen console (Fig. 1) and echoed to a time‐stamped log.txt file stored in the file output directory. Feature files and the LCM are individually analyzed by the Application after which the user is invited to specify valid classes or value ranges. BioSARN uses primitive number arrays (with low software overhead) to reduce compute times. Use of a 64‐bit operating system with a significant RAM is preferable. BioSARN ran the *A. fraseri* Scenario 7 example (discussed later) with an Intel 2.4 GHz Core i‐7 processor in under 2 min.

### Scenarios

There are eight scenarios. BioSARN selects the only possible scenario from user input.

Complete scenario descriptions, FAQS, and data error/issue handling information are available to the user from the “HELP” button on the main screen.

Under scenario 1, the user combines one or more *feature* files with a *baseline* species ENM. Feature files are data used to trim the ENM output to better approximate the species' realized distribution (e.g., edaphic and topographic features). The choice of baseline ENM is at the modeler's discretion. This is distinguished from the projected ENM (used in other BioSARN scenarios) which corresponds to the ENM used by MAXENT's projection facility (Phillips et al. 2004). Scenario 1 output consists of ENM input values exceeding the user‐defined environmental suitability threshold and meeting user‐specified feature file values. Otherwise, output grid cell values are zeroed. Extent and resolution are identical to the ENM.

Scenario 2 computes temporal corridors from input baseline and projected ENMs applying the user‐specified environmental suitability thresholds. Output data values are either 0, 1, 2, or 3 (unsuitable, valid for baseline climate only, valid for both climates, and valid for the projected climate only).

Table 1 summarizes the input file combination and output ASCII file grid cell values for each of seven BioSARN scenarios.

**Table 1:** BioSARN scenario file input combinations and output grid values

BioSARN Scenarios input/output								
Scenario	Input ASCII files				Output ASCII grid values			
	Baseline ENM	Projected ENM	Feature files	LCM	Original ENM	0, 1, 2, 3^a^	Highest+1^a^	Highest+1^a^ Highest+2 Highest+3
1	✓		✓		✓			
2	✓	✓				✓		
3	✓	✓	✓			✓		
4	✓			✓			✓	
5	✓		✓	✓			✓	
6	✓	✓		✓				✓
7	✓	✓	✓	✓				✓

0 = Unsuitable climate; 1 = baseline climate suitability; 2 = both climates suitability; 3 = projected climate suitability.

a The following supplemental values are added to the LCM: Highest+1 LCM value = baseline climate suitability; Highest+2 LCM value = both climates suitability; Highest+3 LCM value = projected climate suitability.

Scenario 8 is a quantification analysis of the input LCM (which can be any ASCII file consisting of categorical data). This analysis can be performed solely on the LCM or the LCM and up to 225 user‐specified tiles. Information including category counts and area quantification is output to an Excel file. Additionally, a shape file grid is output corresponding to the geographic tile‐specified coordinates in the Excel file. Area calculations consider the latitude/longitude of the LCM.

### Example: approximate realized niche of the Fraser fir in the USA

**Figure 2:**
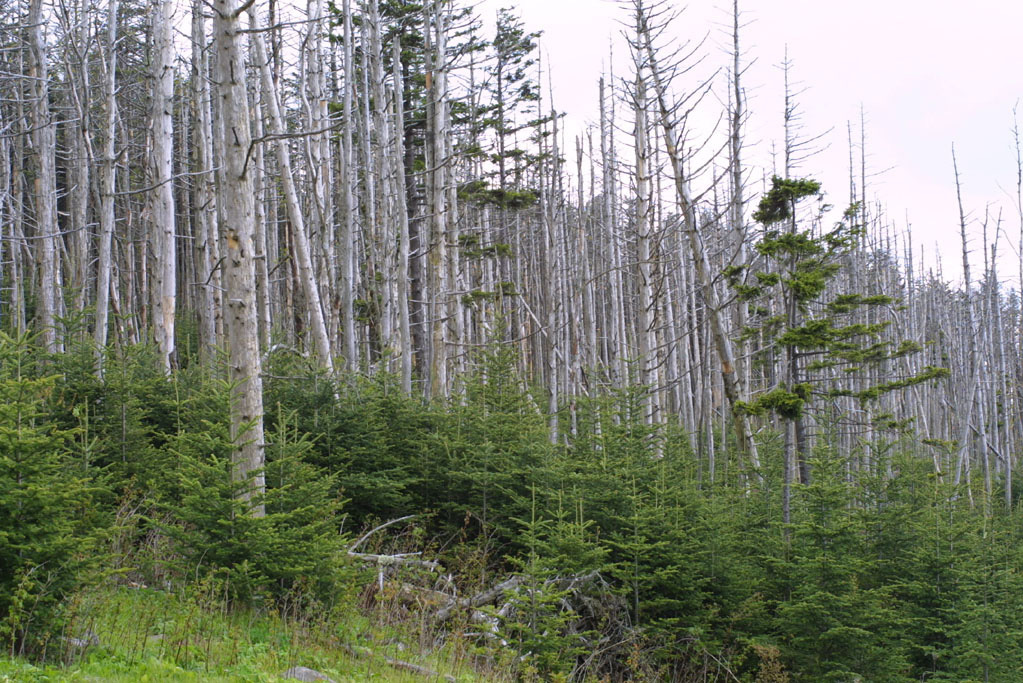
Fraser fir seedlings persist among trunks of trees killed decades earlier at Clingmans Dome, Great Smoky Mountains National Park, Swain County, North Carolina, USA (35.56° latitude, −83.50° longitude). ^©^Steven J. Baskauf http://bioimages.vanderbilt.edu/baskauf/11439.

Fraser fir, *A. fraseri* (Pursh) Poir., is a small evergreen coniferous tree native to the Appalachian Mountains in southeastern USA (Fig. 2). Its preference for extremely acid soils and forested locations make *A. fraseri* an ideal candidate plant species for BioSARN testing.

#### Data and methods

##### Species occurrence data sourcing and cleaning

About 1148 species occurrence data records for *A. fraseri* were downloaded from recognized data repositories – 818 from BISON and 330 from GBIF (BISON 2016; GBIF.org 2016). These data were then cleaned as follows:

- 409 records removed with nonspatially referenced occurrences.
- 27 records removed with coordinates stated <2 decimal places (Heap and Culham 2010).
- 57 records removed with no temporal reference.
- 225 records removed with temporal references outside the 1980–2010 baseline climate period.
- 221 records removed with spatially duplicated references.
- 2 records removed from protruding spatially marginal locations (Soley‐Guardia et al. 2016).
- 2 records removed with erroneously transposed coordinates.

The remaining 205 records were then filtered for locational accuracy. Sixty‐two records were removed with unstated coordinate precision and two records removed with stated coordinate precision >1000 m.

##### Sampling bias mitigation

A systematic sampling filter (Fourcade et al. 2014) was then applied to the remaining 141 occurrences to maintain a minimum 3‐km distance between points, and thus reduce sampling bias (Phillips et al. 2009; Boria et al. 2014). The resulting species occurrence dataset consisted of 27 points. The Appalachian Mountain areas where *A. fraseri* is found are limited in size, and a 3‐km filter was the maximum that could be used to provide a reasonable number of occurrence points for modeling purposes.

##### Environmental layers

The environmental datasets used each consisted of 19 Bioclim layers at 30‐arc seconds resolution.

Baseline climate PRISM monthly ppt, *t*_max_, and *t*_min_ data were downloaded for the coterminous USA (ASCII file format) covering the 30‐year period from 1981 to 2010 (PRISM Climate Group, Oregon State University 2014). Steps 5–9 of Ramírez and Cabrera's methodology (Ramírez‐Villegas and Bueno‐Cabrera 2009) were followed to generate 19 Bioclim layers in DIVA‐GIS from this data. Ramírez and Cabrera's paper is freely available at http://ccafs-climate.org/downloads/docs/Ramirez_Bueno-Cabrera_2009_tutorial_bcvars_creation.pdf.

The MIROC5 model is closest to the multimodel mean (MMM) of the 19 available IPCC 5^th^ Assessment ensemble models for the 2050 (2035–2065 average) projected North American climate (Harris et al. 2014 recommend use of the MMM where possible). The 8.5 RCP emissions scenario was used due to the currently high global warming rate. Nineteen MIROC5 Bioclim layers were downloaded from WorldClim (WorldClim – Global Climate Data 2015).

##### ENM construction details

In a study of several ENM applications, Hernandez et al. (2006) found that MAXENT had the highest accuracy and spatial concordance for small sample size categories. Consequently, baseline and 2050 projected climate models were built with the latest version of MAXENT (3.3.3k). The same user‐defined training and test data were used for all MAXENT models by partitioning 75% of the occurrence data for training (20 points) and 25% for testing (7 points) as used by Phillips et al. (2006). Data partitioning point selection was carried out randomly. Erroneous predictions of suitable habitat under the future climate scenario were avoided using the MAXENT “fade‐by‐clamping” option to remove heavily clamped pixels from the final predictions. Predictions of climate conditions outside the limits encountered during training were constrained by disabling the extrapolation option (Phillips et al. 2006). The MAXENT default setting of 10,000 background points was used.

##### ENM parameterization and evaluation

Multiple MAXENT parameterization scenarios (Muscarella et al. 2014; Soley‐Guardia et al. 2016) were run to obtain the best model. First, four MAXENT models were run using linear (L), quadratic (Q), and hinge (H) feature classes (FCs) appropriate for sample sizes from 15 to 79 occurrence points (Phillips and Dudík 2008). These models adopted the L, LQ, H, LQH feature combinations used by Muscarella et al. (2014) and a regularization multiple (RM) of 1.0.

AUC_diff_ (AUC_train_ − AUC_test_) a threshold independent measure was 0 (0.999 − 0.999) for all models except for the H model where it was −0.001.

Four further models were run based on the LQH model (automatically selected by MAXENT) but with RMs of 2.0, 3.0, 4.0, and 5.0. The RM acts in concert across all FCs as a coefficient multiplied to the individual regularization values (betas in MAXENT) that correspond to each respective FC (Phillips and Dudík 2008). The LQH model with a 3.0 RM was chosen as the best model because the omission rate plot was closest to the predicted omission (Phillips et al. 2006) although any of the computed models could have been used for this study due to their similarity. The environmental suitability threshold used for subsequent BioSARN models was the average of the lowest presence threshold of 0.088 and 10 percentile training presence of 0.473 (i.e., 0.2805) as used by Boria et al. (2014).

##### Construction of environmental layers for use with BioSARN

A soil layer and land cover classification model (LCM) were constructed for use with BioSARN. STATSGO soil data compiled by the Natural Resources Conservation Service of the U.S. Department of Agriculture were used to create the soil layer (STATSGO 2015). The topmost (0–5 cm) of the 11 standard layers defined by Miller and White (1998) was used.

The National Land Cover Database 2011 (henceforth referred to as NLCD 2011) is a decision‐tree classification of circa 2011 Landsat satellite data (Homer et al. 2015). NLCD 2011 has a 16‐class USA land cover classification and 30‐m spatial resolution. The LCM for the Appalachians was clipped from this layer.

##### BioSARN methodology

**Figure 3:**
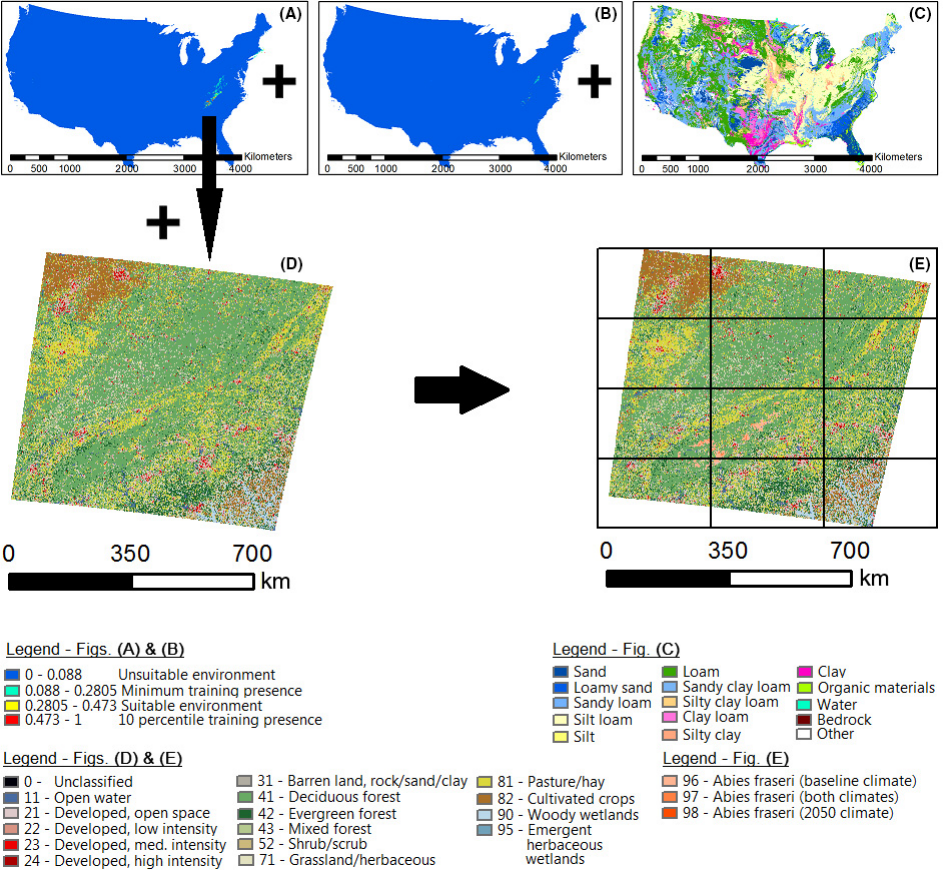
Illustration of BioSARN methodology for realized niche approximation of *Abies fraseri* – (A) MAXENT baseline climate, (B) MAXENT 2050 climate, (C) Soil model, (D) LCM, (E) BioSARN Scenario 7 model with BioSARN Scenario 8 grid overlay. Tile numbering is left/right, top/bottom. Note: The NLCD 2011 LCM has an Albers Conical Equal Area projection.

Two BioSARN Scenario 6 and two Scenario 7 models were constructed and all output ASCII files subject to Scenario 8 quantification analysis to determine the effects of soil and LCM filtering on MAXENT‐generated climatic niches. STATSGO class 6 (loam) was used for soil filtering as all 27 occurrence points occurred in loam areas. LCM filtering was carried out with land class values of 41, 42, and 43 (deciduous forest, evergreen forest, and mixed forest) as these were the classes applying to the 27 occurrence data points used. The overall BioSARN methodology is illustrated in Figure 3.

## Results

**Figure 4:**
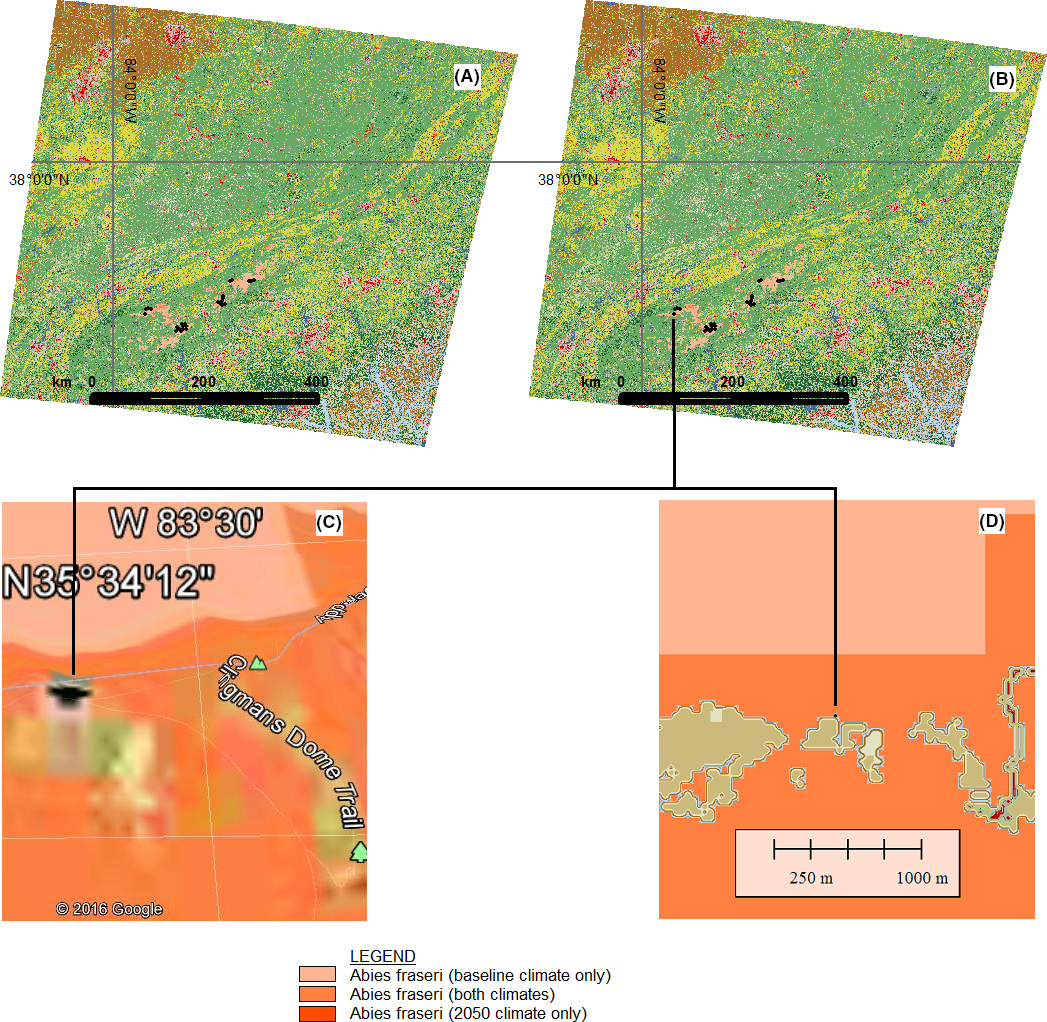
BioSARN realized niche approximation for *Abies fraseri* – (A) BioSARN Scenario 6 distribution with zero filtering and the occurrence point data overlay, (B) BioSARN Scenario 7 distribution with Soil and LCM class filtering, (C) Google Earth Zoom‐in on Clingmans Dome with BioSARN 7 overlay, (D) GlobalMapper/BioSARN 7 view of Clingmans Dome.

Three Abies distribution classes were added to the NLCD 2011 LCM used, namely baseline climate only (96), continuous climate (97), and 2050 climate only (98). Coloring ranged from pale to dark orange (see Fig. 3D legend). Columns 2 and 5 of Table 2 quantify the land class areas in Figure 4A and B.

**Table 2:** BioSARN Scenario 8 quantification analysis summary

Land class	BioSARN Scenario 6		BioSARN Scenario 7	
	MAXENT (km^2^)	LC (km^2^)	Soil (km^2^)	LC, Soil (km^2^)
0	0	0	0	0
11	6,082	6,084	6,082	6,084
21	28,238	28,399	28,250	28,399
22	11,394	11,409	11,395	11,409
23	4,124	4,130	4,125	4,130
24	1,512	1,513	1,512	1,513
31	1,924	1,931	1,925	1,931
41	214,155	214,155	214,362	214,362
42	30,205	30,205	30,243	30,243
43	11,719	11,719	11,741	11,741
52	8,645	8,723	8,659	8,723
71	17,597	17,617	17,598	17,617
81	68,813	68,990	68,846	68,990
82	30,339	30,339	30,339	30,339
90	10,306	10,308	10,306	10,308
95	682	682	682	682
96	3,693	3,255	3,428	3,050
97	305	278	305	278
98	75	71	10	9
Sum 0–98	449,808	449,808	449,808	449,808
Sum 96–98	4,073	3,604	3,743	3,337

The approximate *realized* niche of *A. fraseri* was calculated to decline 91% from 3328 km^2^ (3050 km^2^ + 278 km^2^) to 287 km^2^ (278 km^2^ + 9 km^2^) between the 1980–2010 baseline and 2035–2065 projected climate scenarios (Table 2, column 5). Projected niche change is characterized by a major contraction of the current niche with loss of the southern extension (Table 3, tiles 6–11).

**Table 3:** BioSARN Scenario 8 tile area quantification analysis (Fig. 3e)

Land class area	BioSARN Scen. 7 (LC, Soil) Land Class Tile Area		
	96 (km^2^)	97 (km^2^)	98 (km^2^)
Tile 1	0	0	0
Tile 2	0	0	0
Tile 3	0	0	0
Tile 4	0	0	0
Tile 5	0	0	0
Tile 6	1	0	4
Tile 7	550	93	0
Tile 8	1,924	185	5
Tile 9	0	0	0
Tile 10	374	0	0
Tile 11	201	0	0
Tile 12	0	0	0
Total area	3,050	278	9

The *potential* MAXENT distribution under the baseline climate was 3998 km^2^ (Table 2, column 2). Soil and land class filtering caused this area to decline 17% to 3328 km^2^ (Table 2, column 5), with a 6% contribution from soil and 11% from the LCM (Table 2, column 4).

## Discussion

The use of soil and LCM filtering showed how BioSARN can further refine an ENM *potential* distribution to arrive at a better approximation of a species' *realized* niche. The addition of three output classes provided enhanced temporal corridor definition permitting the observer to distinguish current, continuing, and future niches, and thus niche change and movement (Fig. 4). Tile quantification analysis provided further corroboration of these trends (Table 3). Land class area calculations are fairly accurate as the BioSARN Scenario 8 algorithm utilizes a mathematical equation factoring‐in curvature of the Earth's surface to measure the latitudinal/longitudinal extent, and thus the average grid cell area of the LCM (772 m × 799 m in the example).

Close scrutiny of Figure 4D reveals that the Clingmans Dome occurrence point while in a forested land class category borders a developed open space area. The location of species occurrences is subject to data uncertainty demonstrating the importance of human expertise (e.g., gained via on site observation) in determining appropriate land classes for this species. Unfortunately, this endemic tree species is on the IUCN Red List of Threatened Species^™^ http://www.iucnredlist.org/details/32101/0 and the 91% realized niche contraction projected by this study will only exacerbate the situation. On the bright side, Figure 4C and D indicates that reproductive populations of the Fraser fir should remain at Clingmans Dome for at least the next 50 years or so.

Lastly, when interpreting the results, the limitations of the ENM spatial resolution used should be considered. At 800 m, ENMs cannot identify microclimates occurring within a grid cell nor can they distinguish occurrence points belonging to remnant nonreproductive populations.

These examples illustrate that ENMs are tools that should be used in conjunction with an ecologist's knowledge. BioSARN was developed with this integration in mind.

## Conclusions

The Fraser fir results obtained met the stated aim of the BioSARN application. This approach toward species *realized* niche refinement does not substitute other established ENM methods. Rather, it allows the experimenter to work with their preferred ENM, refining it using their knowledge and experience.

Output from lower spatial resolution ENMs to a high spatial resolution LCM generates a pseudo high‐resolution result. Nevertheless, this is probably the best that can be achieved until wide range high spatial resolution environmental and accurate high precision species occurrence data become generally available.

## Conflict of Interest

None declared.

## Supporting information


**Appendix S1.** BioSARN_Area_Statistics_Scenario7_soil_lc.xls
